# Kinetic constraints on self-assembly into closed supramolecular structures

**DOI:** 10.1038/s41598-017-12528-8

**Published:** 2017-09-25

**Authors:** Thomas C. T. Michaels, Mathias M. J. Bellaiche, Michael F. Hagan, Tuomas P. J. Knowles

**Affiliations:** 1 Department of Chemistry, University of Cambridge, Lensfield Road, Cambridge, CB2 1EW UK; 2 Paulson School of Engineering and Applied Sciences, Harvard University, Cambridge, MA 02138 USA; 3 Laboratory of Chemical Physics, National Institute of Digestive and Diabetes and Kidney Diseases, National Institutes of Health, Bethesda, MD 20892 USA; 4 Department of Physics, Brandeis University, Waltham, MA 02454 USA; 5 Cavendish Laboratory, Department of Physics, University of Cambridge, J J Thomson Avenue, Cambridge, CB3 1HE United Kingdom

## Abstract

Many biological and synthetic systems exploit self-assembly to generate highly intricate closed supramolecular architectures, ranging from self-assembling cages to viral capsids. The fundamental design principles that control the structural determinants of the resulting assemblies are increasingly well-understood, but much less is known about the kinetics of such assembly phenomena and it remains a key challenge to elucidate how these systems can be engineered to assemble in an efficient manner and avoid kinetic trapping. We show here that simple scaling laws emerge from a set of kinetic equations describing the self-assembly of identical building blocks into closed supramolecular structures and that this scaling behavior provides general rules that determine efficient assembly in these systems. Using this framework, we uncover the existence of a narrow range of parameter space that supports efficient self-assembly and reveal that nature capitalizes on this behavior to direct the reliable assembly of viral capsids on biologically relevant timescales.

## Introduction

The spontaneous formation of nanoscale materials with specific chemical and physical characteristics from basic molecular building blocks is a key process for the functioning of living systems and provides a bottom-up strategy for constructing novel nanomaterials for various applications^1^. A particularly important class of such molecular self-assembly processes is the formation of closed supramolecular structures, with examples including clathrin assemblies^2^, self-assembling cages^3,4^, micellar-like structures^5^, small polyhedra^6–8^ or icosahedral viral capsids^9–11^. Many assembly processes of this type underlie key events in normal biology^12^, but are also implicated in the onset of diseases of humans, animals and plants. Moreover, the construction of such molecular topologies offers great potential as biomimetic nanocontainers for encapsulation, delivery and release of small molecules.

Elegant physical principles have emerged that determine the geometric and equilibrium constraints governing the shapes of the resulting assembly structures in these systems, motivating the question of whether or not analogous principles can be defined for their assembly kinetics. Probing molecular reaction mechanisms in complex systems represents a fundamental challenge through the Chemical Sciences; in this context, chemical kinetics has proven to be an extremely effective tool for testing mechanistic hypothesis in areas ranging from small molecule chemistry to enzyme kinetics. Recent advances have extended the applicability of this chemical kinetics approach to the study of filamentous protein assembly phenomena, such as amyloid formation^13,14^, providing fundamental insights into the nature of the microscopic steps in the aggregation process^15–18^. These advances have been made possible by the discovery of integrated rate laws that allow relating experimental measurements to the underlying microscopic mechanisms and hence studying the self-assembly into open-ended fibrillar structures at a highly detailed level^15–18^. It has however remained challenging to exploit the full power of the chemical kinetics approach beyond fibril formation to probe the molecular-level mechanisms of the more complex phenomenon of self-assembly into closed supramolecular structures, a difficultly originating in large part from the absence of integrated rate laws describing such processes. Here, we make a step forward in this direction by deriving a closed-form solution to a set of rate equations describing the assembly kinetics of molecular building blocks into closed target structures^19,20^, and show how the availability of this integrated rate law uncovers, from a kinetic analysis of experimental data, general dynamic constraints on the microscopic rate constants that control efficient supramolecular self-assembly in such systems.

## Results and Discussion

### Fundamental kinetic equations

**Figure 1:**
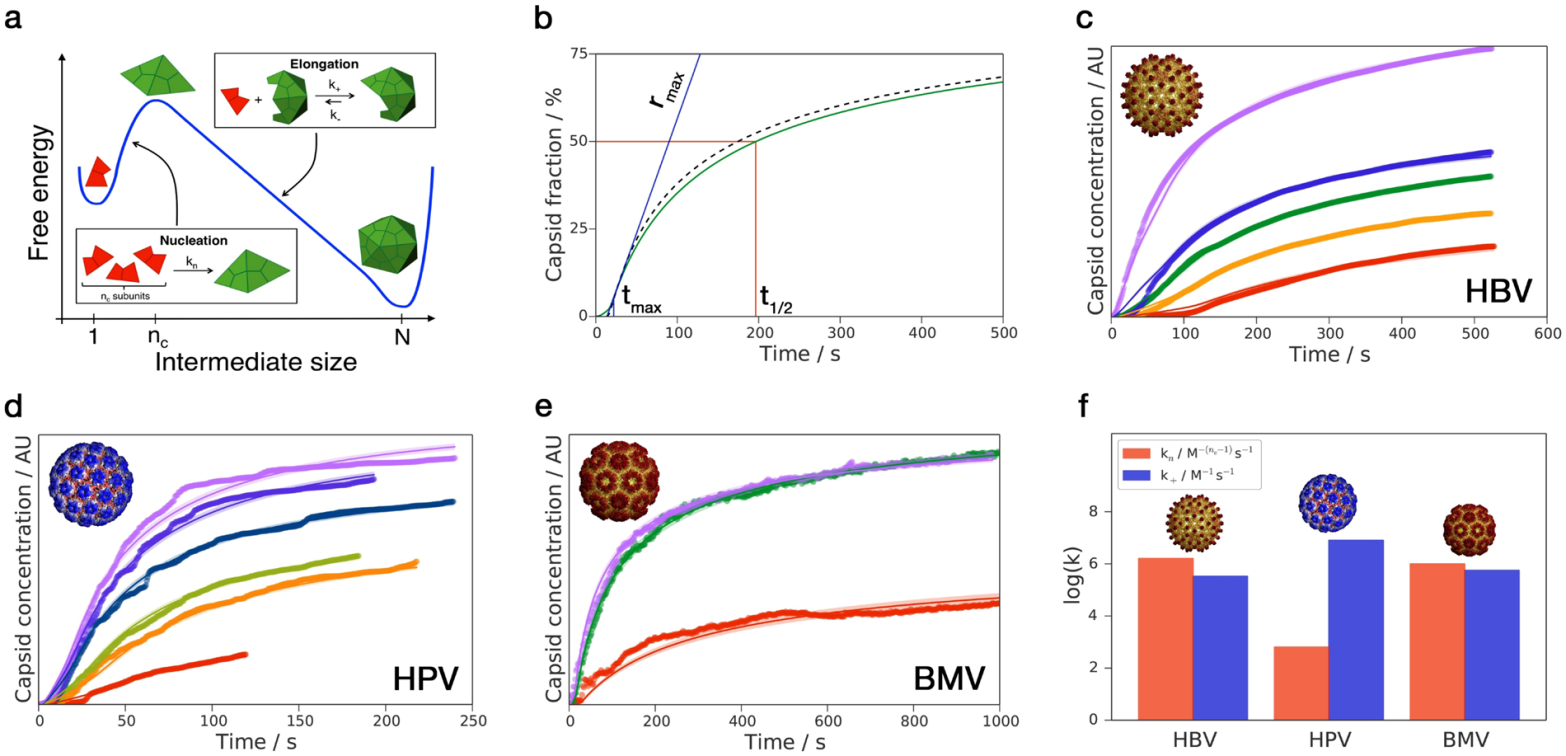
(**a**) Schematic representation of assembly line model: subunits nucleate first and then proceed downhill through elongation reactions to the final structure. Structures in the scheme exemplify assembly with *n*_*c*_ = 3 and *N* = 30. (**b**) Comparison of numerical solution to Eqs (1) and (2) (dashed black) with Eq. (9) (solid blue) and definitions of characteristic times *t*_*max*_ and *t*_1/2_. Calculation parameters: *N* = 90, *n*_*c*_ = 3, *k*_*n*_ = 1 × 10^6^ M^−2^ s^−1^ *k*_+_ = 5.6 × 10^5^ M^−1^ s^−1^ and $m\mathrm{(0)}$ = 10 *μ*M. (**c**–**e**) Global fits of various virus kinetics. (**c**) Hepatitis B Virus with *m*(0) = 3.8, 5.4, 6.4, 8.2 and 10.8 *μ*M. Data from^19^. (**d**) Human Papillomavirus with *m*(0) = 0.40, 0.41, 0.53, 0.72, 0.74, and 0.80 *μ*M. Data from^21^. (**e**) Brome Mosaic Virus with *m*(0) = 6.2, 11.1 and 14.0 *μ*M. Data from^22^. (**f**) Extracted elongation and nucleation rate constants for all viral systems considered. Note that all experimental data analyzed in this work were obtained using purified proteins. Viral images reproduced from^23^ with permission.

The self-assembly of molecular building blocks into closed target structures may be captured by the following set of kinetic equations for the concentration *f*(*t*, *j*) of intermediates of size *j*, known as the assembly line model (Fig. 1(a))^19,20^:

1 $$\begin{array}{rcl}\frac{\partial f(t,j)}{\partial t} & = & {k}_{+}m(t)f(t,j-\mathrm{1)}-{k}_{+}m(t)f(t,j)+{k}_{n}m{(t)}^{{n}_{c}}{\delta }_{j,{n}_{c}}\\ \frac{\partial f(t,N)}{\partial t} & = & {k}_{+}m(t)f(t,N-\mathrm{1),}\end{array}$$

where *N* is the number of subunits in the target structure and *m*(*t*) is the concentration of free subunits in solution, as determined by conservation of the total subunit concentration

2 $$\frac{dm(t)}{dt}=-\frac{d}{dt}\sum _{j={n}_{c}}^{N}\,jf\,(t,j\mathrm{).}$$

The terms on the first line of Eq. (1) describe the growth of assembly intermediates through the addition of individual subunits with rate constant *k*_+_. The term ${k}_{n}m{(t)}^{{n}_{c}}$ describes the initial nucleation step^24^ as the spontaneous formation of the smallest growth-competent intermediate from the interaction of *n*_*c*_ subunits with rate constant *k*_*n*_. Thus, the parameter *n*_*c*_ corresponds to the reaction order of the nucleation step and, in the simplest scenario, can be thought of as the size of the smallest stable assembly intermediate (*n*_*c*_ is the analogous quantity to the critical nucleus size in classical nucleation theory); intermediates with size *j* < *n*_*c*_ are unstable and quickly dissociate back to free subunits, such that their concentration can be assumed to be negligible. Finally, the last equation of (1) describes the end step of the assembly line as the closure of an intermediate of size *N* − 1 into the final structure. Note that in the limit of infinite *N*, Eq. (1) recover the kinetic equations commonly used to describe filamentous protein assembly processes^14–16^. Note that, in Eq. (1) we have assumed size-independent rate constants; this assumption was primarily made for minimizing the number of model parameters to avoid over-fitting in the analysis of kinetic data, but our framework can be extended straightforwardly to take this effect into account. Moreover, Eq. (1) are deterministic and neglect therefore the potential effect of statistical number fluctuations. Such fluctuations are often negligible in reactions in bulk, but can become dominant in reactions under volume confinement^25^. We also note that Eq. (1) assume that assembly results in a single closed capsid geometry. Application to reactions that yield multiple capsid morphologies, such as the CCMV systems studied in refs^26,27^, require a more complex master equation.

### Integrated rate law for assembly kinetics

To obtain an integrated rate law to Eqs (1) and (2), we make use of the perturbative renormalization group (RG)^28^, a general mathematical technique for constructing approximative solutions to nonlinear differential equations. In our case, the applicability of this method relies on the observation that the dimensionless ratio $\varepsilon \equiv {k}_{n}m{\mathrm{(0)}}^{{n}_{c}-2}/{k}_{+}\ll 1$ is a small parameter, where $m\mathrm{(0)}$ is the initial subunit concentration. In order to take advantage formally of the smallness of *ε*, it is convenient to rewrite Eq. (1) in dimensionless form

3 $$\begin{array}{rcl}\frac{\partial \varphi (\tau ,j)}{\partial \tau } & = & \mu (\tau )\varphi (\tau ,j-\mathrm{1)}-\mu (\tau )\varphi (\tau ,j)\\  &  & +\varepsilon \mu {(\tau )}^{{n}_{c}}{\delta }_{j,{n}_{c}},\end{array}$$

where $\tau ={k}_{+}m\mathrm{(0)}t$, $\mu (\tau )=m(\tau )/m\mathrm{(0)}$ and $\varphi (\tau ,j)=f(\tau ,j)/m\mathrm{(0)}$. Solving Eq. (3) perturbatively yields, after some algebra, the following result for the time-varying subunit concentration:

4 $$\begin{array}{rcl}\mu (\tau ) & = & \rho -\varepsilon N{\rho }^{{n}_{c}}\tau \\  &  & +\varepsilon {\rho }^{{n}_{c}-1}[{{\rm{\Omega }}}_{0}-{e}^{-\rho \tau }\sum _{k\mathrm{=0}}^{N-{n}_{c}-1}{{\rm{\Omega }}}_{k}\frac{{\rho }^{k}{\tau }^{k}}{k!}]+\cdots ,\end{array}$$

where $\rho =1$ and ${{\rm{\Omega }}}_{k}=(N-{n}_{c}-k)(N-{n}_{c}-k+\mathrm{1)/2}$. While Eq. (4) is accurate for short times, we observe the emergence of a divergent term $(-\varepsilon N\tau$) at later times, which prevents this linearized early-time solution from being valid over the full time course of the reaction. At a fundamental level, this divergence emerges due to our ignorance about the system’s behavior in the future; in fact, while the information about the initial concentration of subunits is sufficient for describing the system dynamics over short timescales, at later times the lack of information about the way in which *ρ* varies with changing timescale is what causes Eq. (4) to depart from the true solution. Perturbative RG provides a systematic method for dealing with this undesired divergence and hence obtain a global approximation valid for the duration of the whole reaction. Note that this procedure mirrors very closely the conventional RG approaches of quantum field theory and condensed matter physics. In these theories, we are interested in describing how a certain quantity of interest, such as the charge or the mass of an electron, is renormalized as we vary the observation scale (e.g. momentum or energy scale in quantum field theory). The missing information about the large-scale (e.g. high-energy) behavior of the system is packed into so-called counter terms, which are constructed in order to cancel the divergencies in the theory. In our case, the analogous quantity to the electron charge or mass of quantum field theory is the initial concentration of monomers and the RG procedure should yield renormalized values for this quantity at different time scales. Following the conventional work-flow of RG, we start by introducing an arbitrary time scale *σ* which we will vary between the initial time 0 and the observation time *τ* and then allow for a *σ*-dependence of the initial subunit concentration by writing $\rho =\rho (\sigma )+\varepsilon \delta \rho (\sigma )$, where *ρ*(*σ*) is the renormalized subunit concentration (at scale *σ*) and $\delta \rho (\sigma )$ is a counter term. The counter term $\delta \rho (\sigma )=N{\rho }^{{n}_{c}}\sigma$ removes the divergent term in Eq. (4) and so we arrive at the following renormalized expansion

5 $$\mu (\tau )=\rho (\sigma )-\varepsilon N{\rho }^{{n}_{c}}(\tau -\sigma )+ {\mathcal R} ,$$

where ${\mathcal R}$ stands for regular terms. As a next step in the RG framework, we require $\partial \mu /\partial \sigma =0$ since *σ* is arbitrary. Doing so, we arrive at the following RG equation

6 $$\frac{\partial \rho }{\partial \sigma }=-\varepsilon N\rho {(\sigma )}^{{n}_{c}}\mathrm{.}$$

By solving Eq. (6) and substituting in Eq. (5) as $\sigma \to \tau$ we obtain the uniformly valid solutions

7 $$\mu (\tau )=\rho (\tau )+\varepsilon \rho {(\tau )}^{{n}_{c}-1}[{{\rm{\Omega }}}_{0}-{e}^{-\rho (\tau )\tau }\sum _{k\mathrm{=0}}^{N-{n}_{c}-1}{{\rm{\Omega }}}_{k}\frac{\rho {(\tau )}^{k}{\tau }^{k}}{k!}],$$

8 $$\varphi (\tau ,j)=\varepsilon \rho {(\tau )}^{{n}_{c}-1}[1-{e}^{-\rho (\tau )\tau }\sum _{k\mathrm{=0}}^{j-{n}_{c}}\frac{\rho {(\tau )}^{k}{\tau }^{k}}{k!}]\mathrm{.}$$

Finally, using conservation of total subunit concentration and transforming back to real time *t* we arrive at the following integrated rate law for the concentration of closed target structures:

9 $$\begin{array}{rcl}f(t,N) & = & \frac{m\mathrm{(0)}-\rho (t)}{N}-\frac{(N-{n}_{c}){k}_{n}\rho {(t)}^{{n}_{c}-1}}{{k}_{+}}\\  &  & \times [1-{e}^{-{k}_{+}\rho (t)t}\sum _{k\mathrm{=0}}^{N-{n}_{c}-1}\frac{(N-{n}_{c}-k)}{(N-{n}_{c})}\frac{{[{k}_{+}\rho (t)t]}^{k}}{k!}],\end{array}$$

where

10 $$\rho (t)=\frac{m\mathrm{(0)}}{{[1+N({n}_{c}-\mathrm{1)}{k}_{n}m{\mathrm{(0)}}^{{n}_{c}-1}t]}^{\mathrm{1/(}{n}_{c}-\mathrm{1)}}}\mathrm{.}$$

This solution shows overall good agreement with the numerical evaluation of Eqs (1) and (2) (Fig. 1(b) and see Supplementary Material for a discussion on the accuracy of Eq. (9) as a function of *ε*). Moreover, we note that within the first-order RG approximation discussed here the kinetic trace for capsid formation is systematically underestimated by the analytical solution. This is because the function *ρ*(*t*) obtained by solving the first-order RG equation decays faster than the true solution. These errors can be reduced by applying the RG method to higher orders in *ε*.

### General characteristics of assembly kinetics

Using the integrated rate law, Eq. (9), we are now in the position to derive, from first principles, a number of relationships characterizing the time course of the assembly reaction. According to Eq. (9), the time evolution of the concentration of target structures demonstrates the characteristic sigmoidal shape defined by an initial lag phase followed by a phase of rapid growth and final asymptotic approach to the plateau^20^. A defining feature of the early time behaviour is the presence of a point of inflection *t*_*max*_ at which the growth rate *r* = *df*(*t*, *N*)/*dt* is maximal. Solving the equation $dr/dt{|}_{{t}_{max}}=0$ yields the position of the inflection point as

11 $${t}_{max}=\frac{N-{n}_{c}}{{k}_{+}m\mathrm{(0)}}\mathrm{.}$$

The time of inflection is determined completely by the characteristic elongation timescale (*k*_+_ *m*(0))^−1^. The physical interpretation of Eq. (11) is that of the time required for *N *−* n*_*c*_ elongation steps to occur. This result is consistent with the idea that the lag phase of the reaction corresponds to a waiting period during which the assembly line is set up and all intermediate states are populated^20^.

The maximal growth rate ${r}_{max}=df(t,N)/dt{|}_{{t}_{max}}$ is computed from Eq. (9) as

12 $${r}_{max}=\frac{{k}_{n}m{\mathrm{(0)}}^{{n}_{c}}{(N-{n}_{c})}^{N-{n}_{c}}}{(N-{n}_{c})!}{e}^{-(N-{n}_{c})}\mathrm{.}$$

Note that *r*_*max*_ is given by the product of the rate of rate-limiting nucleation step and the Poissonian probability of observing the minimal number *N* − *n*_*c*_ of elongation steps required to complete the assembly structure. A key prediction of Eq. (12) is the emergence of a power-law scaling of the maximal growth rate with initial subunit concentration ${r}_{max} \sim m{\mathrm{(0)}}^{\gamma }$. Because the scaling exponent *γ* solely depends on the nature of the nucleation step, *γ* = *n*_*c*_, the critical nucleus size can be determined from the slope of a log-log plot of *r*_*max*_ vs *m*(0). Thus, as in many other areas of science^29,30^, scaling laws emerge in the context of supramolecular assembly as a general property that connects macroscopic data with the physical nature of the underlying microscopic processes through the value of the scaling exponent.

Equation (9) implies that the median assembly time *t*_1/2_, defined by the condition $f({t}_{\mathrm{1/2}},N)=m\mathrm{(0)/(2}N)$, is given by ${t}_{\mathrm{1/2}}=2{t}_{max}+{t}_{nuc}$, where

13 $${t}_{nuc}=\frac{{2}^{{n}_{c}}-1}{N({n}_{c}-\mathrm{1)}{k}_{n}m{\mathrm{(0)}}^{{n}_{c}-1}}$$

is the time needed to consume half the free subunits after the assembly line is set up, assuming that each nucleation event leads to the target structure through the eventual consumption of *N* subunits (see Supplementary Information). This result shows that the *t*_1/2_ is given as a sum of two distinct contributions, one originating from *t*_*max*_, the time necessary to form a quasi-steady state of intermediates, and the other from *t*_*nuc*_ + *t*_*max*_, the time to nucleate a sufficient amount of intermediates that mature into the final structure through the the chain reactions of the assembly line. The former contribution to *t*_1/2_ depends only on the efficiency of the elongation reactions in the assembly line, while the latter is governed by nucleation events. Crucially, the relative importance of these two contributions to the median assembly time is determined by the parameter $\varepsilon ={k}_{n}m{\mathrm{(0)}}^{{n}_{c}-2}/{k}_{+}$. This quantity–which measures the ratio of the rates of nucleation and elongation–naturally emerges from our theoretical framework as the key parameter controlling the assembly kinetics. In general, large values of *ε* correspond to a kinetic trap, whereby subunits are significantly depleted by nucleating too quickly, leaving less material to complete the assembly of target structures. By contrast, when *ε* is small, few nuclei are formed and the assembly yield is low for relevant time scales. The crossover between these two regimes occurs when *t*_*nuc*_ = 2*t*_*max*_. Using the results above, this criterion can be formulated as a condition on the parameter *ε* as:

14 $${\varepsilon }_{c}=\frac{{2}^{{n}_{c}-1}-1}{2N(N-{n}_{c})({n}_{c}-\mathrm{1)}}\mathrm{.}$$

When *ε* > *ε*_*c*_, the system is susceptible to kinetic traps, whereas when *ε* < *ε*_*c*_ the assembly is inefficient. According to this criterion, successful assembly is the result of a delicate balance between the necessity of forming appreciable amounts of target structures and the danger of being kinetically trapped. Controlling the relative importance of nucleation and elongation processes provides therefore a high degree of intrinsic regulation of self-assembly^20^. We note that *ε*_*c*_ decreases with increasing size *N* of the target geometry as *N* ^−2^. This behavior follows intuition because larger target structures impose stronger constraints on the time available for nucleation, *t*_*nuc*_ ~ 1/*N*, while the time required for producing the quasi-steady state assembly line, *t*_max_ ~ *N*, is inevitably longer for larger *N*.

### Kinetic analysis of experimental data

**Figure 2:**
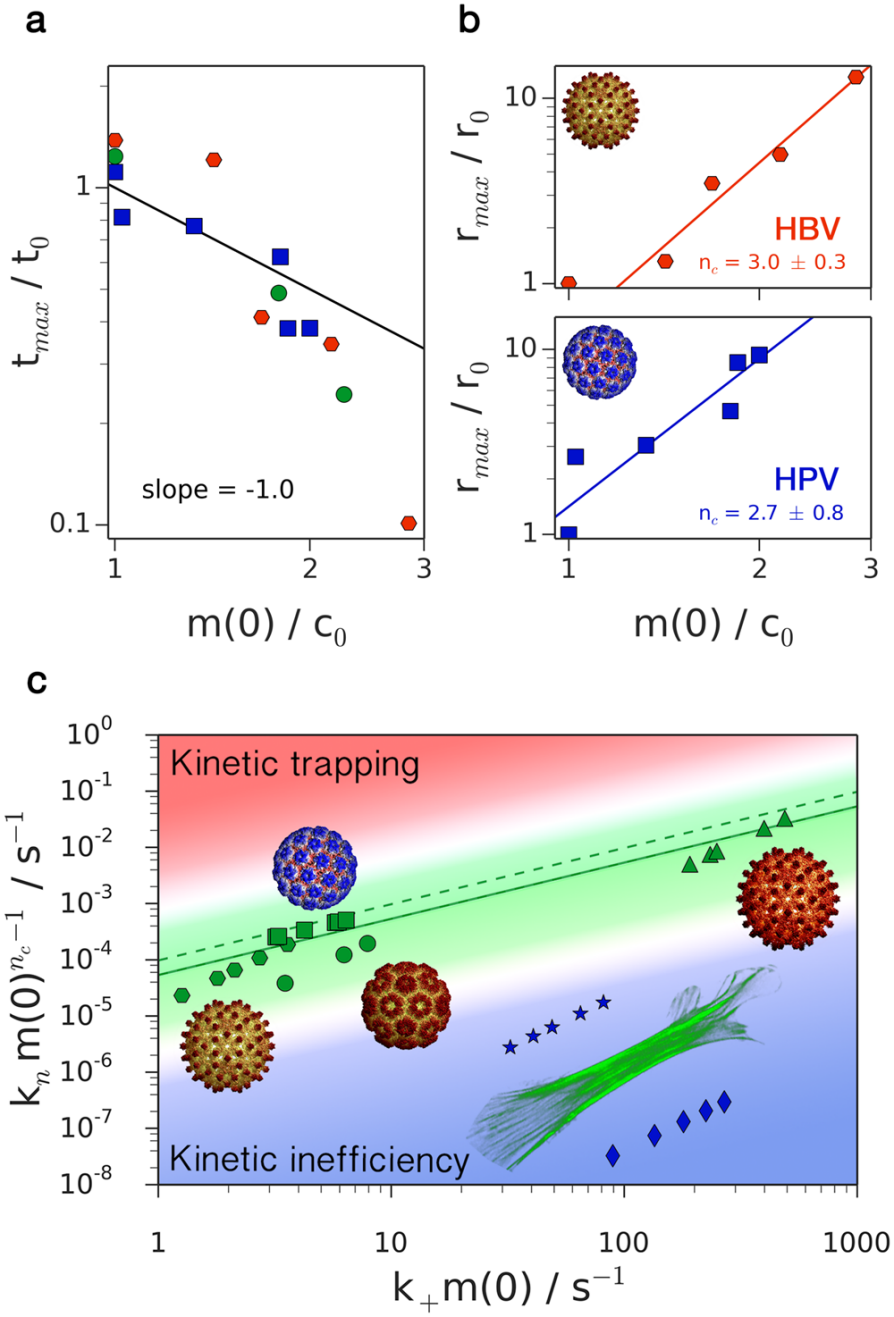
Scaling behavior of viral capsid assembly properties and assembly efficiency. (**a**) Scaling of *t*_*max*_ with *m*(0) as predicted by Eq. (11) for various viral systems with data shown as circles (BMV), squares (HPV) and hexagons (HBV). (**b**) The reaction order for nucleation, *n*_*c*_, is obtained from the scaling behavior of *r*_*max*_. The data are for HBV and HPV. Note that discontinuities in the experimental kinetic traces for HPV assembly are responsible for inaccuracies in determining *r*_*max*_ for data at higher initial concentrations. (**c**) Balance between elongation and nucleation in the viral systems studied in this work. Green solid line corresponds to ε = 4.9 × 10^−5^, green dashed lines correspond to *ε*_*c*_ (Eq. (14)) for the various viruses. BMV data denoted by circles, HPV by squares, HBV by hexagons and HBV assembly data obtained at 37 °C and pH 7.5 from^50^ (see Supplementary Information) by triangles. Blue data are from actin polymerization measurements in magnesium (stars) or in calcium (diamonds) from^51^. Viral images reproduced from^23^ with permission.

Through the analysis of experimental kinetic data, we now demonstrate that the theoretical framework provided by Eq. (9) is capable of describing macroscopic features of supra-molecular self-assembly into closed topologies in terms of microscopic rate constants. We took a representative example and considered kinetic data of the formation of icosahedral viral capsids. Since the current version of our theory only considers empty capsid assembly, we limit our comparison to *in vitro* experiments on the assembly of purified capsid proteins; i.e., the systems do not include viral genomes, other viral proteins, or host factors. Previous studies modeling viral capsid assembly kinetics using master equations^19,20,31–36^, continuum models^37–39^ or molecular dynamics simulations^40–49^ have led to important insights into the system characteristics, yet it remains a key challenge to elucidate the general physical principles underlying capsid assembly. First, we consider the assembly kinetics of Human Hepatitis B Virus (HBV)^19^, a representative icosahedral virus comprised predominantly of *N* = 120 subunits. Figure 1(c) shows the time evolution of HBV capsid concentration, as monitored by light scattering intensity, fit globally to the integrated rate law Eq. (9) with fixed *n*_*c*_ = 3 (as determined from the scaling of maximal growth rate, Fig. 2(b)), yielding rate constants of ${k}_{+}=3.32\pm 0.15\times {10}^{5}$ M^−1^ s^−1^ and ${k}_{n}=1.6\pm 0.9\times {10}^{6}$ M^−2^ s^−1^. The global nature of the fit demonstrates the consistent agreement between Eq. (9) and the full time courses observed in the experiment over a wide range of initial subunit concentrations, including the characteristic sigmoidal shape of kinetic traces. We note that the entire data set could be fitted to Eq. (9) using just two global rate constants and one concentration-dependent plateau parameter for each kinetic curve that accounts for the constant of proportionality between the measured light scattering signal and the capsid concentration. In the SI, we provide also fitting to HBV assembly data under reducing conditions at 37 °C and pH 7.5 from ref.^50^. The fits to these higher temperature data, however, are less accurate, which could arise due to late stage intermediates^52^ and protein interconversion between assembly-active and assembly-inactive conformations^53,54^. Next, we consider kinetic data for the formation of Human Papillomavirus (HPV, Fig. 1(d))^21^ and Brome Mosaic Virus (BMV, Fig. 1(e)) capsids^22^. Using *n*_*c*_ = 2, *N* = 72 (HPV) and *n*_*c*_ = 3, *N* = 90 (BMV), global fits of experimental data to Eq. (9) with *k*_+_ = 8.0 ± 0.5 × 10^6^ M^−1^ s^−1^, ${k}_{n}=6.4\pm 0.4\times {10}^{2}$ M^−1^ s^−1^ (HPV) and ${k}_{+}=5.6\pm 1.0\times {10}^{5}$ M^−1^ s^−1^, ${k}_{n}=9.9\pm 0.8\times {10}^{6}$ M^−2^ s^−1^ (BMV) are again able to describe the full time course of the assembly reactions. Furthermore, from the analysis of the experimental kinetic data, we can also directly verify the scaling predictions that have resulted from our analytical treatment of the master equation (1) for the three virus systems discussed here. Figure 2(a) shows a double logarithmic plot of the measured inflection times, *t*_*max*_, against initial subunit concentration for the three systems considered in Fig. 1 together with the predicted scaling law, ${t}_{max} \sim m{\mathrm{(0)}}^{-1}$. The scatter in the data for the inflection time is due to increased experimental noise in the kinetic profiles close to the initial point of the reaction. Moreover, Fig. 2(b) illustrates how the relevant value for the reaction order for the nucleation step, *n*_*c*_, can be determined from the analysis of the maximal growth rate as a function of total subunit concentration. Through the analysis of the maximal growth rate, it is therefore possible to fix the value of *n*_*c*_ necessary for fitting kinetic traces. We note that the value of *n*_*c*_ for BMV was set to 3 as reported previously in the literature^22^; this was done because the corresponding dataset has too few points for confident fitting. We also note that similar scaling laws have been previously obtained approximately by assuming an ad hoc separation between nucleation and growth processes^39,55^.

The availability of microscopic rate constants enabled by the present analysis allows mechanistic comparisons to be made between the assembly of different virus capsid systems. Interestingly, while the absolute values of the rate constants obtained from the fitting of experimental data vary over several orders of magnitude (Fig. 1(f)), we observe that the parameter *ε* takes similar values across the different data sets: $\varepsilon =5.0\pm 0.4\times {10}^{-5}$ ($m\mathrm{(0)}=10\,\mu$M) for HBV, $\varepsilon =8.0\pm 0.7\times {10}^{-5}$ ($m\mathrm{(0)}=1\,\mu$M) for HPV and $\varepsilon =1.7\pm 0.3\times {10}^{-5}$ ($m\mathrm{(0)}=10\,\mu$M) for BMV. Moreover, these values fall in the same order of magnitude as the theoretical predictions for ${\varepsilon }_{c}$: $5.5\times {10}^{-5}$ (HBV), $1.0\times {10}^{-4}$ (HPV) and $9.5\times {10}^{-5}$ (BMV). This illustrates how the apparently distinct viral systems studied in this work are characterized by a similar balance of the relative rates of elongation and nucleation to achieve successful assembly. By contrast, for filamentous protein self-assembly^14–16^ the long-time average length of aggregates $\langle L\rangle$ becomes $\langle L\rangle  \sim \mathrm{1/}\sqrt{{\varepsilon }_{c}}$. As linear systems such as actin^51^ are required by their biological function to be long, they should have low measured *ε* so as to maximize efficiency. This prediction is in agreement with what is observed in Fig. 2(c).

## Conclusions

In conclusion, although it is of both fundamental and practical interest to identify and characterize the kinetic constraints governing supra-molecular self-assembly into closed target structures, this understanding has proved challenging to achieve in practice. Here, we have demonstrated how the availability of integrated rate laws to the underlying kinetic equations illuminates the dynamic design criteria that characterize the efficiency of such processes. We showed that efficient assembly only occurs in a narrow range of parameter space. By applying this kinetic analysis to experimental data of icosahedral viral capsid assembly we demonstrated that these structures occupy this narrow region of parameter space corresponding to efficient assembly.

## Electronic supplementary material

Supplementary Information
