# Supplementary material for: Kinetic constraints on self-assembly into closed supramolecular structures
Source: Sci Rep. 2017 Sep 25;7:12295. doi: 10.1038/s41598-017-12528-8 (PMC5613031; doi:10.1038/s41598-017-12528-8)
Supplement: Supplementary file 1 — Supplementary Information [file 41598_2017_12528_MOESM1_ESM.pdf]

Supplementary Information to “Kinetic constraints on self-assembly into closed supramolecular structures”

July 17, 2017

Thomas C. T. Michaels[1,2], Mathias M. J. Bellaiche[1,3], Mike F. Hagan[4] & Tuomas P. J. Knowles[1,5,*]

[1]Department of Chemistry, University of Cambridge, Lensfield Road, Cambridge, CB2 1EW, UK,
[2]Paulson School of Engineering and Applied Sciences, Harvard University, Cambridge, MA 02138, USA
[3]Laboratory of Chemical Physics, National Institute of Digestive and Diabetes and Kidney Diseases, National Institutes of Health, Bethesda, MD 20892, USA
[4]Department of Physics, Brandeis University, Waltham, MA 02454, USA,
[5]Cavendish Laboratory, Department of Physics, University of Cambridge, J J Thomson Avenue, Cambridge, CB3 1HE, United Kingdom.
*To whom correspondence should be addressed; E-mail: tpjk2@cam.ac.uk.

1 Additional HBV assembly data

Data were also obtained for HBV assembly under reducing conditions at 37°C and pH 7.5 from Ref. [1] (these data are referred to here as HBV$_\Delta$). The extracted kinetic parameters were $k_n = 3.87 \pm 0.04 \times 10^8$ M^{-2}s^{-1} and $k_+ = 5.31 \pm 0.09 \times 10^7$ M^{-1}s^{-1}. Fits are shown in Fig. 1.

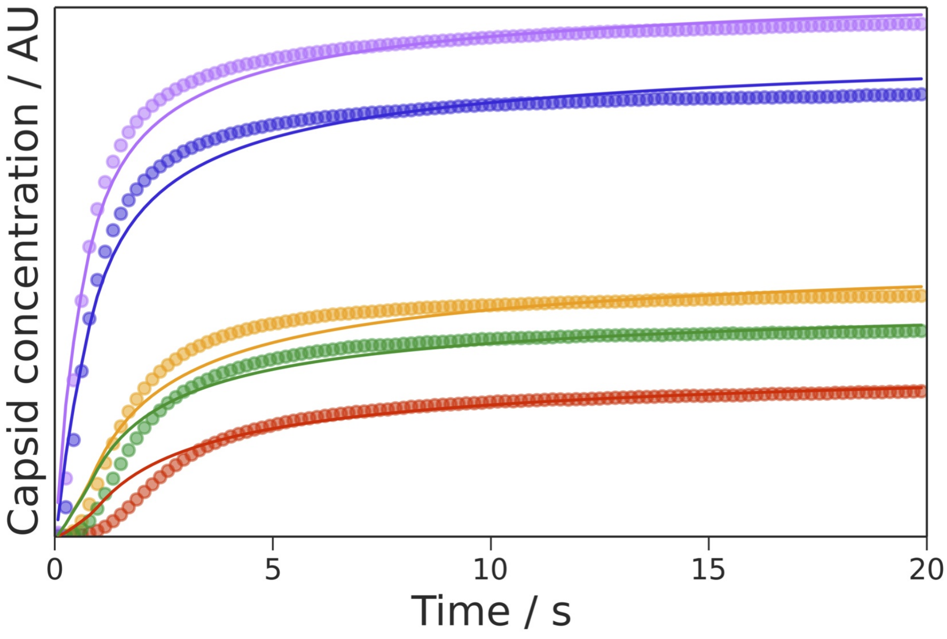

Figure 1: Global fit to HBV_{Δ} assembly data for $m(0)$ = 3.6, 4.4, 4.7, 7.5 and 9.2 μM.

2 Data analysis

2.1 Fitting

The analytical solution presented in the main manuscript (Eqs. (9) and (10) of the main text) was used to fit experimental data globally. Fits were performed by minimizing χ^2, the total square deviation between the experimental data and the analytical prediction:

$$\chi^2 = \sum_l \sum_i \left(\frac{y_i - c_l f(t_i, \boldsymbol{p})}{\sigma_i} \right)^2, \tag{1}$$

with the l sum runs over the different kinetic traces contributing to the global fit, the i sum over the data points of the lth trace, y_i the measurement at time t_i, σ_i the error in measurement y_i (if available), $\boldsymbol{p}$ the collection of fit parameters, f the function given by Eq. (9) of the main text and c_l a individual fit parameter described in more depth later. Eq. (9) of the main text, however, involves a finite sum, which for large values of N becomes expensive computationally to evaluate many times to ensure a good fit. To overcome this problem, the properties of the

incomplete gamma function $\Gamma(s, x) = \int_x^\infty e^{-t} t^{s-1} dt$ were used to rewrite Eq. (9) of the main text as follows:

$$
\begin{aligned}
f(t, N) =& \frac{1}{N}\Big(m(0) - m(t) - \frac{k_n}{k_+}\rho(t)^{n_c-1}\Big[\frac{(N-n_c)(N+n_c-1)}{2} - e^{-x}x^{N-n_c+1}\frac{N+n_c+x}{2(N-n_c)!} \\
&+ \frac{N^2 - N - 2n_c x - n_c^2 + n_c - x^2}{2}\frac{\Gamma(N-n_c+1, x)}{\Gamma(N-n_c+1)}\Big]\Big), \qquad (2)
\end{aligned}
$$

where $x = k_+ m(t) t$ and

$$
\begin{aligned}
m(t) =& \rho(t) + \frac{k_n}{k_+}\rho(t)^{n_c-1}\Big[\frac{(N-n_c)(N-n_c+1)}{2} - e^{-x}x^{N-n_c+2}\frac{N-n_c-x-1}{2(N-n_c+1)!} \\
&- \frac{N^2 + n_c^2 - 2Nx + 2n_c x + x^2 + N - n_c - 2Nn_c}{2}\frac{\Gamma(N-n_c+2, x)}{\Gamma(N-n_c+2)}\Big]. \qquad (3)
\end{aligned}
$$

This strategy was done to increase computational speed greatly as tabulated gamma functions were called from the `scipy` module. Minimization of χ^2 was accomplished by using the `basinhopping` algorithm of `scipy`, which is described in Ref. [2] and has been successfully used in the past to fit experimental data of amyloid growth to integrated rate laws of similar complexity [3].

2.2 Goodness of fit

Residuals for all fits presented are shown in Fig. 2. For most systems, there was a large positive residual peak occurring in the early times, before t_{max}. This is due to small truncation errors in many $(N - n_c)$ intermediate concentrations being propagated by mass conservation to errors in the capsid concentration. Doing a perturbation expansion of $\phi(t, j)$ to higher orders in ε would resolve these errors.

Regardless, no system except for HBV_Δ showed major systematic residuals for late times, which is diagnostic of a good fit especially considering the rate parameters k_+ and k_n were global parameters. The HBV_Δ fits, however, show major systematic residuals, meaning that

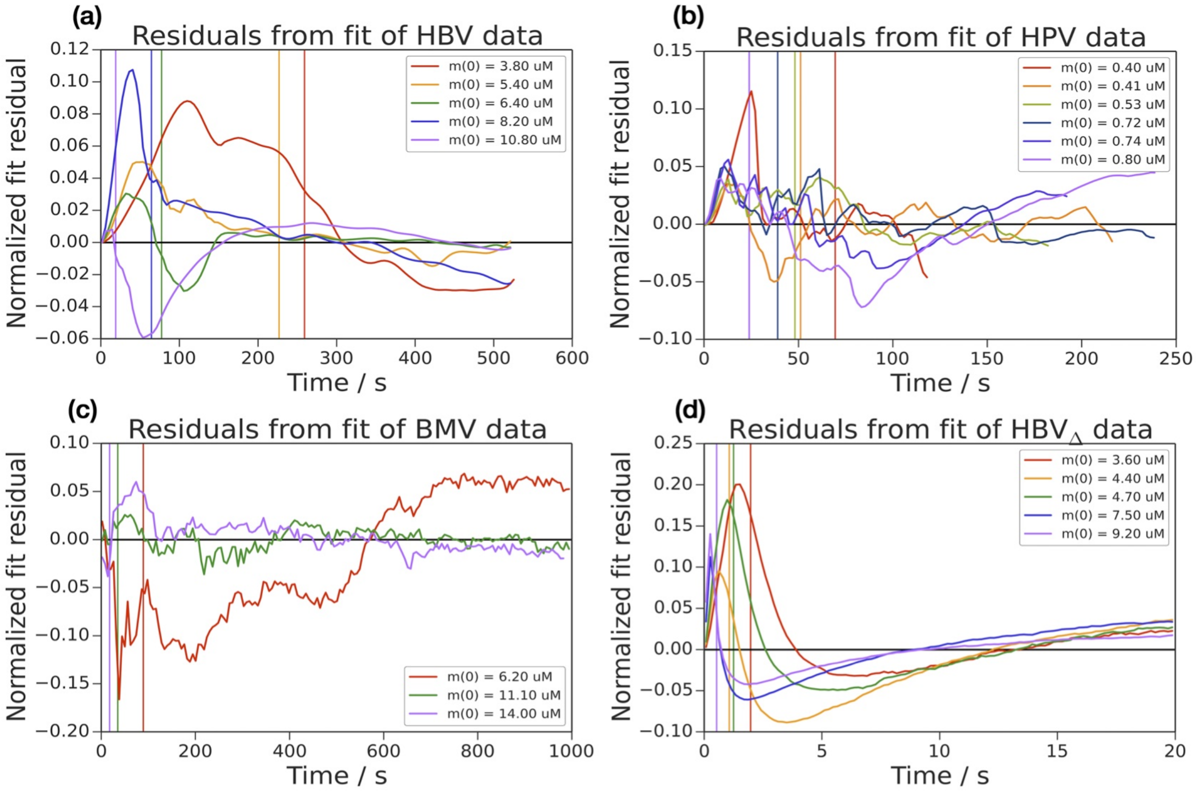

Figure 2: **(a)-(d)** Resiudals of global fits for all data shown.

our model was somehow too simplistic to capture the full behavior of this system's assembly. In particular, we attributed the systematic errors to late stage intermediates[7] and protein interconversion between assembly-active and assembly-inactive conformations.[8, 9]

Regardless of the errors mentioned, it should be stressed that our fits use the minimum number of fitting parameters (one for each rate constant and one for each initial concentration-dependent plateau value) to describe the data. While the plateau value should scale in a predictable fashion with $m(0)$, this parameter (c_l in Eq. (1)) was nevertheless kept an individual fit parameter instead of a global one to allow for experimental error between traces and uncertainties as to the zero-point of the reactions due to dead time between mixing reagents and starting measurements to be absorbed into one throwaway constant. Furthermore, as this is a multiplicative constant, it will only affect the scale of the fit, not the shape, which is determined by the rate constants [10]. Other theoretical models have shown good agreement between with experimental data, for example in Ref. [4], but these models use a far greater number of fit parameters, which mathematically will necessarily give a better fit. Hence, one does not gain as much physical insight into the underlying process by having a complicated model. In our case, we have only two free parameters controlling the shape of the fit, which allows us to extract the maximum possible physical information from the available data.

2.3 Error analysis

Experimental errors for the data shown in the main paper were unavailable, so actually our fit implicitly assumed each datum carried the same error. The error in each datum point was approximated *a posteriori* by noticing that for a perfect fit, $\chi^2 = \sum_i \left(\frac{y_i - f(x_i, \boldsymbol{p})}{\sigma}\right)^2$ tends toward N_{dof}, the number of degrees of freedom (i.e. the number of data points minus the number of fit parameters). Hence we can approximate $\sigma \sim \sqrt{\frac{\left(\sum_i y_i - f(x_i, \boldsymbol{p})\right)^2}{N_{\text{dof}}}}$. This approximation was done for all fits shown in the main paper, and this approximate error was propagated to errors in the

fit parameters, thereby yielding the reported rate constant errors.

Errors in the fit parameters were calculated by constructing the covariance matrix of the fit function given by Eq. 2 of the main text with respect to the fit parameters collected in the vector $\boldsymbol{p}$. The covariance matrix about the fit parameters $\underline{C}(\boldsymbol{p})$ is given by:

$$\underline{C}(\boldsymbol{p}) = \sigma^2 \underline{\nabla}_p f(\boldsymbol{p}) \left(\underline{\nabla}_p f(\boldsymbol{p})\right)^T , \tag{4}$$

where $\underline{\nabla}_p f(\boldsymbol{p})$ denotes the gradient of $f(\boldsymbol{p})$ (Eq. 2 of the main text multiplied by c_l) with respect to the fit parameters. In constructing the matrix $\underline{C}(\boldsymbol{p})$, derivatives of $f(\boldsymbol{p})$ were weighted by the assumed error σ, wherefore this matrix was a direct measure of error, as opposed to a relative one. The error in p_i was then simply $\sqrt{\underline{C}[i,i]}$ [5]. This construction was done for each individual fit and the reported errors in rate constants are the root-mean-square averages within a global fitting set.

3 Time scale analysis of virus capsid formation

Matched asymptotics analysis [12] provides an alternative strategy to the perturbative renormalization group (RG) approach [13] for studying solutions of the assembly line master equation (Eq. (1) of the main text) [11]. In this section, we apply this technique to identify the key time scales that characterize the assembly process.

3.1 Assembly dynamics in the boundary layer

Introducing the parameter $\varepsilon \equiv k_n m(0)^{n_c-2}/k_+$ and normalised concentrations of free subunits $\mu(\tau) = m(\tau)/m(0)$ and capsid intermediates $\phi(\tau, j) = f(\tau, j)/m(0)$ allows rewriting the

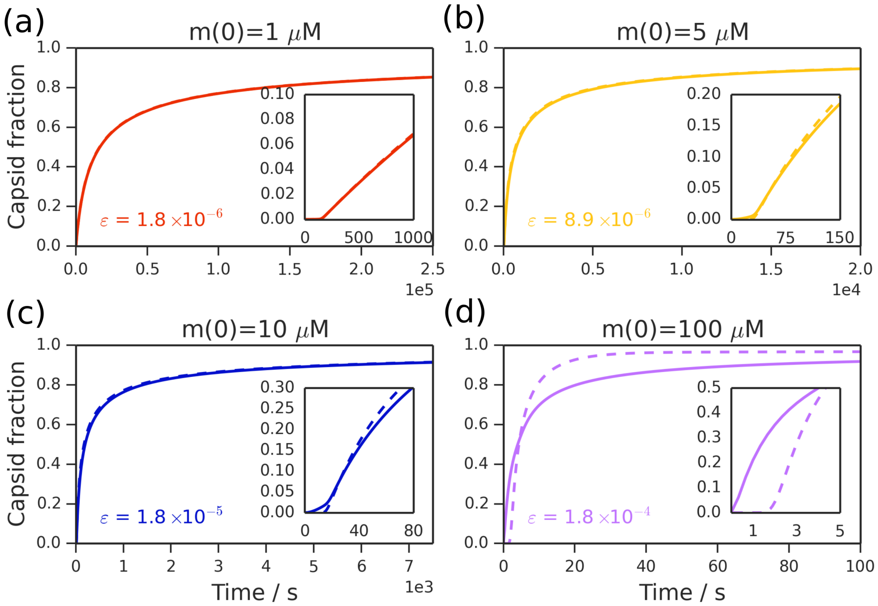

Figure 3: Comparison between RG solution (Eqs. (9) and (10) of the main text, solid lines) and the numerical solution of the master equation (Eqs. (1) and (2) of the main text, dashed lines) for various values of the perturbation parameter $\varepsilon = k_n m(0)^{n_c-2}/k_+$ obtained by varying the total subunit concentration $m(0)$ between 1-100 μM, while leaving the other parameters the same as in Fig. 1b of the main text. As expected, the RG solution becomes increasingly accurate as the perturbation parameter ε tends to zero. The insets highlight the early time behavior; time is in units of 10^5 seconds (a), 10^4 seconds (b), and 10^3 seconds (c).

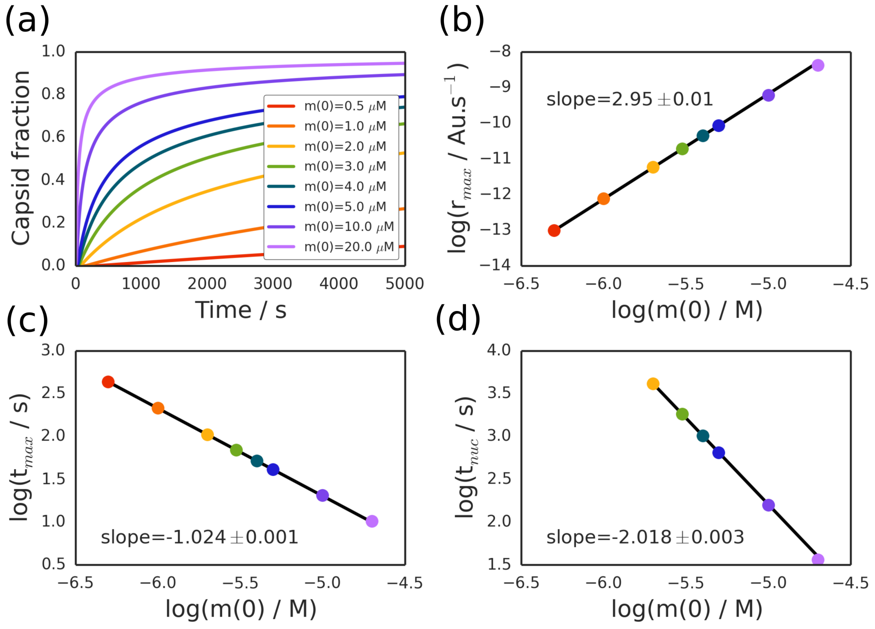

Figure 4: Validation using numerical solutions to the master equation (a) of the scaling relationships for $r_{\max} \propto m(0)^{n_c}$ (b), $t_{\max} \propto m(0)^{-1}$ (c), and $t_{\text{nuc}} \propto m(0)^{-n_c+1}$ (d) obtained in the main text (see Eqs. (11), (12) and (13) of the main text). Calculation parameters as in Fig. 1b of the main text (in particular, $n_c = 3$).

assembly line master equation (Eq. (1) of the main text) as

$$
\begin{aligned}
\frac{\partial\phi(\tau,j)}{\partial\tau} &= \mu(\tau)\phi(\tau,j-1) - \mu(\tau)\phi(\tau,j) + \varepsilon\mu(\tau)^{n_c}\delta_{j,n_c} \\
\frac{\partial\phi(\tau,N)}{\partial\tau} &= \mu(\tau)\phi(\tau,N-1) \\
\frac{d\mu(\tau)}{d\tau} &= -n_c\varepsilon\mu(\tau)^{n_c} - \mu(\tau)\sum_{j=n_c}^{N-1}\phi(\tau,j),
\end{aligned}
\tag{5}
$$

where $\tau = k_+m(0)t$. This dimensionless the assembly line master equation is particularly convenient for understanding the various time scales involved in the growth process.

For $\varepsilon \ll 1$, the reaction initially proceeds through a time scale, in which τ is of order unity. Solving Eq. (5) using the perturbation series

$$
\begin{aligned}
\mu(\tau) &= \mu^{(0)}(\tau) + \varepsilon\mu^{(1)}(\tau) + \mathcal{O}(\varepsilon^2) \\
\phi(\tau,j) &= \phi^{(0)}(\tau,j) + \varepsilon\phi^{(1)}(\tau,j) + \mathcal{O}(\varepsilon^2)
\end{aligned}
\tag{6}
$$

yields $\phi^{(0)}(\tau,j) \equiv 0$, $\mu^{(0)}(\tau) \equiv \rho$ and, therefore,

$$
\begin{aligned}
\frac{\partial\phi^{(1)}(\tau,j)}{\partial\tau} &= \rho\phi^{(1)}(\tau,j-1) - \rho\phi^{(1)}(\tau,j) + \rho^{n_c}\delta_{j,n_c} \qquad (7) \\
\frac{d\mu^{(1)}(\tau)}{d\tau} &= -n_c\rho^{n_c} - \rho\sum_{j=n_c}^{N-1}\phi^{(1)}(\tau,j).
\end{aligned}
$$

Hence, the following regular perturbation result at order ε is obtained for the time evolution of the concentration of intermediates of size j

$$
\phi(\tau,j) = \varepsilon\rho^{n_c-1}\left(1 - e^{-\rho\tau}\sum_{k=0}^{j-n_c}\frac{\rho^k\tau^k}{k!}\right) + \mathcal{O}(\varepsilon^2). \tag{8}
$$

By combining Eq. (8) with Eq. (7) we obtain, upon integration, the following expression for the normalised concentration of free subunits valid up to order ε

$$
\mu(\tau) = \rho + \varepsilon\left\{-N\rho^{n_c}\tau + \rho^{n_c-1}\left[\Omega_0 - e^{-\rho\tau}\sum_{k=0}^{N-n_c-1}\Omega_k\frac{\rho^k\tau^k}{k!}\right]\right\} + \mathcal{O}(\varepsilon^2), \tag{9}
$$

where $\Omega_k = (N - n_c - k)(N - n_c - k + 1)/2$. Finally, the concentration of capsids at order ε is obtained by integrating Eq. (8) for $j = N - 1$, yielding

$$\phi(\tau, N) = \varepsilon \rho^{n_c} \tau - \varepsilon \rho^{n_c - 1}(N - n_c) \left(1 - e^{-\rho\tau} \sum_{k=0}^{N-n_c-1} \frac{(N - n_c - k)}{(N - n_c)} \frac{\rho^k \tau^k}{k!} \right). \tag{10}$$

The above equations (8), (9), (10) recover the initial time behavior characteristic of the situation of a system with constant subunit concentration. In particular, the concentration of nuclei evolves according to a convex exponential profile $\sim 1 - e^{-\rho\tau}$, whereas the concentrations of larger intermediates $j > n_c$ show a sigmoidal profile, as expected due to the multi-step nature of the assembly process. During this initial time scale the concentrations of capsid intermediates vary very rapidly. Mathematically, this phase corresponds to an initial boundary layer in which the solution quickly changes to adjust the boundary conditions in the form of a propagating wave front.

3.2 Assembly dynamics in the slow manifold

After the initial boundary layer, the reaction enters a second time scale (slow manifold), where time is of the order $t \sim [k_n m(0)^{n_c-1}]^{-1}$ or, equivalently, $\tau \sim 1/\varepsilon$. A convenient way of parametrizing the system dynamics along the slow manifold is to introduce a stretched time variable

$$\tau_2 = \varepsilon \tau. \tag{11}$$

By using the perturbation series expansions

$$\mu(\tau_2) = \rho(\tau_2) + \varepsilon \mu^{(1)}(\tau_2) + \cdots, \quad \phi(\tau_2, j) = \varepsilon \phi^{(1)}(\tau_2, j) + \cdots, \quad \phi(\tau_2, N) = \phi^{(0)}(\tau_2, N) + \cdots \tag{12}$$

we obtain the following rate equations

$$\varepsilon^2 \frac{\partial \phi^{(1)}(\tau_2, j)}{\partial \tau_2} = \varepsilon\rho(\tau_2)\phi^{(1)}(\tau_2, j-1) - \varepsilon\rho(\tau_2)\phi^{(1)}(\tau_2, j) + \varepsilon\rho(\tau_2)^{n_c}\delta_{j,n_c} + \cdots \tag{13}$$

$$\varepsilon \frac{\partial \rho(\tau_2)}{\partial \tau_2} = -\varepsilon n_c \rho(\tau_2)^{n_c} - \varepsilon\rho(\tau_2)\sum_{j=n_c}^{N-1}\phi^{(1)}(\tau_2, j) + \cdots$$

Hence, at leading order in ε

$$0 = \rho(\tau_2)\phi^{(1)}(\tau_2, j-1) - \phi^{(1)}(\tau_2, j) + \rho(\tau_2)^{n_c}\delta_{j,n_c} \tag{14}$$

$$\frac{\partial \rho(\tau_2)}{\partial \tau_2} = -n_c\rho(\tau_2)^{n_c} - \rho(\tau_2)\sum_{j=n_c}^{N-1}\phi^{(1)}(\tau_2, j).$$

The first equation implies that

$$\phi^{(1)}(\tau_2, N-1) = \phi^{(1)}(\tau_2, N-2) = \cdots = \phi^{(1)}(\tau_2, n_c) = \rho^{n_c-1}(\tau_2). \tag{15}$$

Hence, the dynamics of the system takes place on a subspace of the space of all trajectories, in which the temporal evolution of capsid intermediates is dictated by the time evolution of the monomer concentration at leading order (Eq. (15)). By combing Eq. (15) with Eq. (14), we obtain

$$\frac{\partial \rho(\tau_2)}{\partial \tau_2} = -n_c\rho(\tau_2)^{n_c} - \rho(\tau_2)\sum_{j=n_c}^{N-1}\rho(\tau_2)^{n_c-1} = -N\rho(\tau_2)^{n_c} \tag{16}$$

and

$$N\frac{\partial \phi^{(0)}(\tau_2, N)}{\partial \tau_2} = -\frac{\partial \rho(\tau_2)}{\partial \tau_2}. \tag{17}$$

Note that this last equation corresponds to Eq. (11) in Ref. [11]. Most importantly, when expressed in terms of the original variable $\tau = \tau_2/\varepsilon$ Eq. (16) recovers the RG equation described in the main manuscript (Eq. (6) of the main text).

In summary, when the system is close to the slow manifold capsid intermediates are in a dynamic quasi-steady state with their concentrations being all equal at leading order and the dynamics is dictated by the RG equation. Hence, the RG equation describes the system dynamics along the slow manifold.

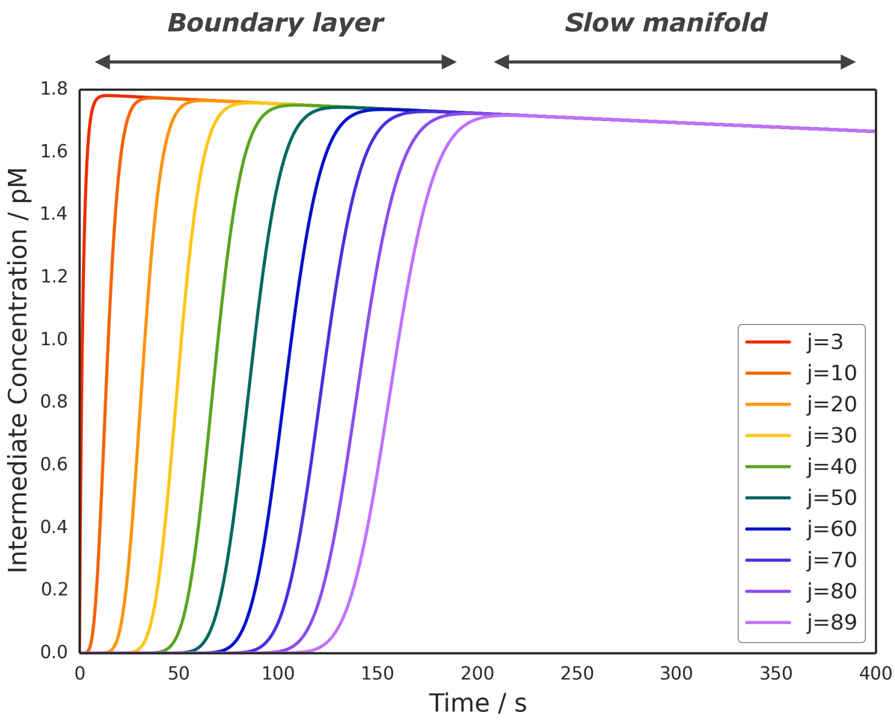

Figure 5: The kinetics of capsid intermediate concentrations is separated into two regimes: an initial boundary layer regime where the assembly line is set up followed by a slow manifold regime where all intermediate concentrations collapse onto a single curve, as described by Eq. (15). The curves were obtained using the BMV parameters (Fig. 1e) and an initial subunit concentration of 1 μM.

3.3 Discussion

Our asymptotic analysis of Eqs. (1) of the main text shows that, when ε is small, the system evolves through two main timescales. Initially, capsids and intermediates are sparsely populated and the system proceeds in the forward direction to set up the assembly line (in the asymptotic analysis literature, this initial phase is termed "boundary layer"). During these early stages of the reaction (typically about 10-20$\%$ of the full reaction time course), there are relative variations in the populations of the various intermediates, as described by SI Eq. (8) and shown in SI Fig. 5. Over longer timescales, however, the kinetic curves for the various intermediate concentrations converge towards a single curve (known as the "slow manifold", SI Eq. (15)), so that at leading order in epsilon intermediates are all equally populated (see SI Fig. 5, which clearly shows the collapse of all intermediate concentrations onto a single curve after about 200 seconds). In this regime, the extent of variations in the intermediate concentrations (i.e. the deviations from the slow manifold) are very small, being bound by $\varepsilon^2 m(0)$.

4 Calculation of half-time

We present here the mathematical details pertaining to the derivation of the formula for the half time $t_{1/2}$ described in the main text. From Eq. (9) of the main text, the rescaled half-time $\tau_{1/2} = k_+ m(0) t_{1/2}$ is obtained as solution of the following equation

$$\frac{1}{2} - \rho(\tau) - \varepsilon \rho^{n_c - 1} \left[N(N - n_c) - e^{-\rho\tau} \sum_{k=0}^{N-n_c-1} N(N - n_c - k) \frac{\rho^k \tau^k}{k!} \right] = 0, \qquad (18)$$

where $\rho(t) = m(0)/\left[1 + N(n_c - 1)\varepsilon\tau\right]^{1/(n_c-1)}$ is the solution of the RG equation (Eq. (6) of the main text). Due to its highly non-linear nature, Eq. (18) cannot be solved exactly. Equation (18), however, is amenable to an approximation scheme based on Newton's method [14]. As the starting point for this approximation scheme, we choose $\tau_{1/2}^{(0)} = \tau_{\text{nuc}} = \frac{2^{n_c-1}-1}{N(n_c-1)\varepsilon}$ [15, 16] and

compute the next order correction using

$$\tau_{1/2}^{(1)} = \tau_{1/2}^{(0)} - \frac{f(\tau_{1/2}^{(0)})}{f'(\tau_{1/2}^{(0)})}, \tag{19}$$

where $f(\tau)$ denotes the left-hand side of Eq. (18). Using the RG equation $\rho' = -\varepsilon N \rho^{n_c}$ (Eq. (6) of the main text) and the fact that $\rho(\tau_{1/2}^{(0)}) = 1/2$ [15], we find

$$\tau_{1/2}^{(1)} = \tau_{1/2}^{(0)} + \frac{2^{1-n_c}\mathcal{Q}}{N2^{-n_c} + N(n_c-1)2^{2-2n_c}\mathcal{Q} + 2^{1-n_c}\left[\frac{1-2^{1-n_c}}{n_c-1} - 1\right](\mathcal{Q} - \mathcal{S} - N(N-n_c))}, \tag{20}$$

where

$$\mathcal{Q} = N(N-n_c) - e^{-\alpha}\sum_{k=0}^{N-n_c-1} N(N-n_c-k)\frac{\alpha^k}{k!} \tag{21}$$

$$\mathcal{S} = e^{-\alpha}\sum_{k=0}^{N-n_c-1} kN(N-n_c-k)\frac{\alpha^{k-1}}{k!} \tag{22}$$

and $\alpha = \tau_{1/2}^{(0)}/2$. Because $\alpha \sim 1/\varepsilon \gg 1$, we neglect exponential decaying terms $e^{-\alpha}$ in Eq. (20) over other terms and arrive at the simple result

$$\tau_{1/2}^{(1)} = \tau_{1/2}^{(0)} + 2(N-n_c). \tag{23}$$

By transforming back to real time $t_{1/2}^{(1)} = \tau_{1/2}^{(1)}/[k_+ m(0)]$, we have

$$t_{1/2}^{(1)} = t_{1/2}^{(0)} + \frac{2(N-n_c)}{k_+ m(0)} = t_{\text{nuc}} + 2t_{\text{max}}, \tag{24}$$

where t_{nuc} and t_{max} are defined in the main text.

References and Notes

[1] L. Selzer, S.P. Katen, A. Zlotnick, The hepatitis B virus core protein intradimer interface modulates capsid assembly and stability. *Biochem* **53**, 5496-5504 (2014).

[2] D.J. Wales, J.P.K. Doye, Global Optimization by Basin-Hopping and the Lowest Energy Structures of Lennard-Jones Clusters Containing up to 110 Atoms. *J Phys Chem A* **101**, 5111-5116 (1997).

[3] G. Meisl *et al*. Differences in nucleation behavior underlie the contrasting aggregation kinetics of the Aβ40 and Aβ42 peptides. *Proc Natl Acad Sci USA* **111**, 9384-9 (2014).

[4] C. Chen, C.C. Kao, B. Dragnea, Self-Assembly of Brome Mosaic Virus Capsids: Insights from Shorter Time-Scale Experiments. *J Phys Chem A* **112**, 9405-9412 (2008).

[5] P.H. Richter, Estimating Errors in Least-Squares Fitting. *TDA Progress Report* **42-122** (1995).

[6] K. Zhou, L. Li, Z. Tan, A. Zlotnick, S.C. Jacobson, Characterization of Hepatitis B Virus Capsids by Resistive-Pulse Sensing. *J Am Chem Soc* **133**, 1618-1621 (2011).

[7] E.E. Pierson, D.Z. Keifer, L. Selzer, L.S. Lee, N.C. Contino, J.C. Wang, A. Zlotnick, and M.F Jarrold, *J. Am. Chem. Soc.* **136**, 3536-41 (2014).

[8] C. Packianathan, S.P. Katen, C.E. Dann 3rd, and A. Zlotnick, *J. Virol.* **84**, 1607-15 (2010).

[9] G.R. Lazaro and M.F. Hagan, *J. Phys. Chem. B* **120**, 6306-18 (2016).

[10] Note that, to leading order, the constants c_l also implicitly account for dissociation effects ($k_- \neq 0$).

[11] D. Endres, A. Zlotnick, Model-based analysis of assembly kinetics for virus capsids or other spherical polymers. *Biophys J* **83**, 1217-1230 (2002).

[12] M. Holmes, *Introduction to Perturbation Methods* (Springer-Verlag, New York, USA, 1995).

[13] L.-Y. Chen, N. Goldenfeld, Y. Oono, Renormalization group theory for global asymptotic analysis. *Phys Rev Lett* **73**, 1311-1315 (1994).

[14] E. Süli, D. Mayers, *An Introduction to Numerical Analysis* (Cambridge University Press, 2003).

[15] The value $\tau_{1/2}^{(0)} = \tau_{\text{nuc}} = \frac{2^{n_c-1}-1}{N(n_c-1)\varepsilon}$ emerges as solution to $\rho(\tau_{1/2}^{(0)}) = 1/2$.

[16] M.F. Hagan, O.M. Elrad, Understanding the Concentration Dependence of Viral Capsid Assembly Kinetics-the Origin of the Lag Time and Identifying the Critical Nucleus Size. *Biophys J* **98**, 1065-1074 (2010).
